# Editorial: Interplay Between RNA Processing Machinery and Epigenetic Regulation in Gene Expression

**DOI:** 10.3389/fgene.2021.799874

**Published:** 2021-12-02

**Authors:** Naoyuki Kataoka, William C. Cho

**Affiliations:** ^1^ Laboratory of Cellular Biochemistry, Department of Animal Resource Sciences, Graduate School of Agriculture and Life Sciences, The University of Tokyo, Tokyo, Japan; ^2^ Department of Clinical Oncology, Queen Elizabeth Hospital, Hong Kong, China

**Keywords:** RNA processing, mRNA, lncRNA, miRNA, epigenetic regulation

In higher eukaryotes, gene expression consists of many steps such as transcription, mRNA processing, RNA transport and translation. Actually these steps are not fully independent, but they affect each other. It has been well accepted that there is the interplay between mRNA splicing and other gene expression steps (Boehm and Gehring, 2016; Schlautmann and Gehring, 2020).

There are accumulating lines of evidence that the epigenetic modifications in DNAs, histones and non-coding RNAs regulate not only RNA processing steps, but also other steps such as cell proliferation. Moreover, the defects in RNA processing and epigenetic regulation could result in many human diseases. Thus, this research topic aims to uncover novel interplay between epigenetics and RNAs in gene expression steps and diseases in human.

Pluripotent stem cells are good models for both application to regenerative medicine and to understand the biological process of embryonic development. Chen et al. focused on the role of lncRNA in stem cell fate through epigenetic regulation. Oct4 and Sox2 are well known transcription factors as members of Yamanaka-factors. They discussed the role of the Oct4 and Sox2 promoter-interacting lncRNA identified by Chromatin RNA *In Situ* reverse Transcription sequencing (CRIST-seq). LncRNAs play important roles in stem cell pluripotency maintenance, differentiation and their dysfunction-related human diseases through different mechanisms. Recently, many lncRNAs were discovered to maintain stem cell pluripotency and determine their lineage differentiation. It was found that the sequence conservation, the RNA structure and the location of those lncRNAs are likely good indicators for their biological functions in cells and organs. Future studies to identify disease-responsible lncRNAs with information for their functions may identify good candidates for therapeutic approaches for some hereditary diseases.

LncRNAs also serve as “sponge” to absorb another functional noncoding RNA, miRNA. Wu et al. characterized the regulatory network among YAP1 and its targeted miRNAs and lncRNAs in Hu sheep SMSCs. YAP/TAZ act in the Hippo pathway, and YAP1 is able to regulate cell proliferation, migration, and apoptosis. While miR-29a significantly reduces the mRNA and protein expression level by binding to a specific 3′-UTR of YAP1, lncRNA CTTN-IT1 restores the expression of YAP1 through competitive binding to miR-29a. Furthermore, CTTN-IT1 can promote the proliferation and differentiation of SMSCs. A CTTN-IT1-miR-29a-YAP1 regulatory network contributes new insights into improving the muscle development of Hu sheep.

There is another “sponge” RNA produced by back-splicing, which is called as circular RNA (circRNA) (Sharma et al., 2021; Verduci et al., 2021). CircRNA plays an important role in tumorigenesis and progression of non-small cell lung cancer (NSCLC). Sun et al. investigated differentially expressed circRNAs and identified the pathogenesis hub genes of NSCLC by comprehensive bioinformatics analysis. They found 10 hub genes, at which the expression of MYLIP, GAN, and CDC27 were related to NSCLC patient prognosis. The relationship among circRNAs-miRNAs-target genes may provide novel insights for unraveling pathogenesis of NSCLC.

The other famous non-coding RNA, microRNA, which is much shorter than lncRNA and mRNA, also have critical roles in many biological steps, such as proliferation and development. Luo et al. described that miR-222 overexpression repressed cell cycle progression and proliferation, and it promoted the apoptosis of immature porcine Sertoli cells. miR-222 directly targeted the 3′UTR of the GRB10 mRNA and reduced its abundance. Their results indicated that miR-222 suppresses immature porcine Sertoli cell growth by targeting the GRB10 gene through inactivation of the PI3K/AKT signaling pathway. This study provided novel insights into the epigenetic regulation of porcine spermatogenesis by determining the fate of Sertoli cells.

Wang et al. investigated the regulatory mechanism of miR-143-3p and lncRNA RP11-363N22.3–functioning upstream of KRAS–in exosomes derived from human mesenchymal stem cells (hMSCs) in pancreatic cancer. The exosomes are novel tools for cell-cell communications that contain RNAs and proteins. It is likely that miR-143-3p regulates KrasG12D, PI3K, ERK, JNK, p38MAPK, and vimentin synergistically to promote apoptosis and suppress cell growth, invasion, and migration in pancreatic cancer.

On the other hand, Wang et al. investigated Renal ischemia–reperfusion injury (IRI) and acute kidney injury (AKI). To determine the biological function and molecular mechanism of action of miR-92a-3p, they assessed the relationship between nuclear factor-erythroid 2-related factor 1 (Nrf1) and TEC pyroptosis induced by ischemia–reperfusion *in vivo* and oxygen–glucose deprivation/reoxygenation (OGD/R). They found that Nrf1 is a potential target for miRNA miR-92a-3p, and the inhibition of miR-92a-3p alleviated oxidative stress *in vitro* and decreased the expression levels of NLRP3, caspase-1, GSDMD-N, IL-1β, and IL-18 *in vitro* and *in vivo*. The results suggested that the inhibition of miR-92a-3p lessen tubular epithelial cell oxidative stress and pyroptosis by targeting Nrf1 in renal IRI.

Lastly, RNA processing is also coordinated with 3′ end processing. The mRNA type RNA including lncRNA undergoes 3′ end processing which is coupled with transcription termination in higher eukaryotes. The majority of eukaryotic genes are subject to alternative polyadenylation (APA) by utilizing multiple poly(A) sites. Soles and Shi summarized APA mechanism with *cis*-regulatory elements and trans-acting factors. In addition, they described important functions for epigenetic mechanisms in APA, including modifications of DNA and histones and higher-order chromatin structures. Although many of epigenetic regulators were shown to play important roles in APA regulation, it is still unclear how they affect RNA polymerase II processivity. Future studies on APA and epigenetics will contribute to identify “adaptors” that connect histones with APA factors to regulate polyadenylation. This will also provide information for the biological consequences of epigenetics-mediated APA regulation in development and in human diseases.

In summary, our research topic focuses on the interplay between biological steps and RNA network presents many interesting associations between epigenetics and RNAs, including miRNA, ceRNA, lncRNA and mRNA. RNA processing steps are not fully independent but linked with other gene expression steps in higher eukaryotes. Future studies to uncover more interplays will contribute to elucidate whole gene expression networks in eukaryotes.
